# The Retina in Multiple System Atrophy: Systematic Review and Meta-Analysis

**DOI:** 10.3389/fneur.2017.00206

**Published:** 2017-05-24

**Authors:** Carlos E. Mendoza-Santiesteban, Iñigo Gabilondo, Jose Alberto Palma, Lucy Norcliffe-Kaufmann, Horacio Kaufmann

**Affiliations:** ^1^Department of Neurology, Dysautonomia Center, New York University School of Medicine, New York, NY, United States; ^2^Biocruces Health Research Institute, Neurodegenerative Diseases Group, Barakaldo, Spain

**Keywords:** multiple system atrophy, retina, alpha-synuclein, optical coherence tomography, ganglion cell layer, retinal nerve fiber layer

## Abstract

**Background:**

Multiple system atrophy (MSA) is a rare, adult-onset, rapidly progressive fatal synucleinopathy that primarily affects oligodendroglial cells in the brain. Patients with MSA only rarely have visual complaints, but recent studies of the retina using optical coherence tomography (OCT) showed atrophy of the peripapillary retinal nerve fiber layer (RNFL) and to a lesser extent the macular ganglion cell layer (GCL) complex.

**Methods:**

We performed a literature review and meta-analysis according to the preferred reporting items for systematic reviews and meta-analyses guidelines for studies published before January 2017, identified through PubMed and *Google Scholar* databases, which reported OCT-related outcomes in patients with MSA and controls. A random-effects model was constructed.

**Results:**

The meta-analysis search strategy yielded 15 articles of which 7 met the inclusion criteria. The pooled difference in the average thickness of the RNFL was −5.48 μm (95% CI, −6.23 to −4.73; *p* < 0.0001), indicating significant thinning in patients with MSA. The pooled results showed significant thinning in all the specific RNFL quadrants, except in the temporal RNFL quadrant, where the thickness in MSA and controls was similar [pooled difference of 1.11 µm (95% CI, −4.03 to 6.26; *p* = 0.67)]. This pattern of retinal damage suggests that MSA patients have preferential loss of retinal ganglion cells projecting to the magnocellular pathway (M-cells), which are mainly located in the peripheral retina and are not essential for visual acuity. Visual acuity, on the other hand, relies mostly on macular ganglion cells projecting to the parvocellular pathway (P-cells) through the temporal portion of the RNFL, which are relatively spared in MSA patients.

**Conclusion:**

The retinal damage in patients with MSA differs from that observed in patients with Parkinson disease (PD). Patients with MSA have more relative preservation of temporal sector of the RNFL and less severe atrophy of the macular GCL complex. We hypothesize that in patients with MSA there is predominant damage of large myelinated optic nerve axons like those originating from the M-cells. These large axons may require higher support from oligodendrocytes. Conversely, in patients with PD, P-cells might be more affected.

## Introduction

Multiple system atrophy (MSA) is a rare, adult-onset fatal synucleinopathy, a group of neurodegenerative disorders driven by abnormal intracellular aggregation of misfolded hyper-phosphorylated fibrillar α-synuclein (αSyn) (1). In MSA, the initial abnormal αSyn deposition occurs in oligodendroglial cells forming glial cytoplasmic inclusions while in other synucleinopathies αSyn deposits occur in neurons forming Lewy bodies and Lewy neurites (2). MSA has common motor and non-motor clinical features with Parkinson disease (PD), but the clinical course of MSA is usually rapid with mean survival below 10 years from diagnosis and with no effective symptomatic or neuroprotective treatments (3–5).

Visual symptoms are not frequent in patients with MSA, but recent studies using optical coherence tomography (OCT) showed progressive retinal thinning with a distinctive pattern and anatomic distribution, which has now been confirmed in postmortem studies (6). Because current candidate biomarkers for MSA from blood, cerebrospinal fluid, and brain or cardiac imaging are neither sensitive nor specific or insufficiently explored (7), OCT-detected retinal abnormalities could emerge as a useful biomarker of disease progression (8). In this article, we briefly review the normal anatomy of the retina and perform a literature review of retinal abnormalities as a biomarker in patients with MSA and a meta-analysis on the main results of OCT studies in patients with MSA. Finally, we discuss putative pathological mechanisms that may explain the observed retinal abnormalities in these patients.

### Anatomy of the Retina

**Figure 1:**
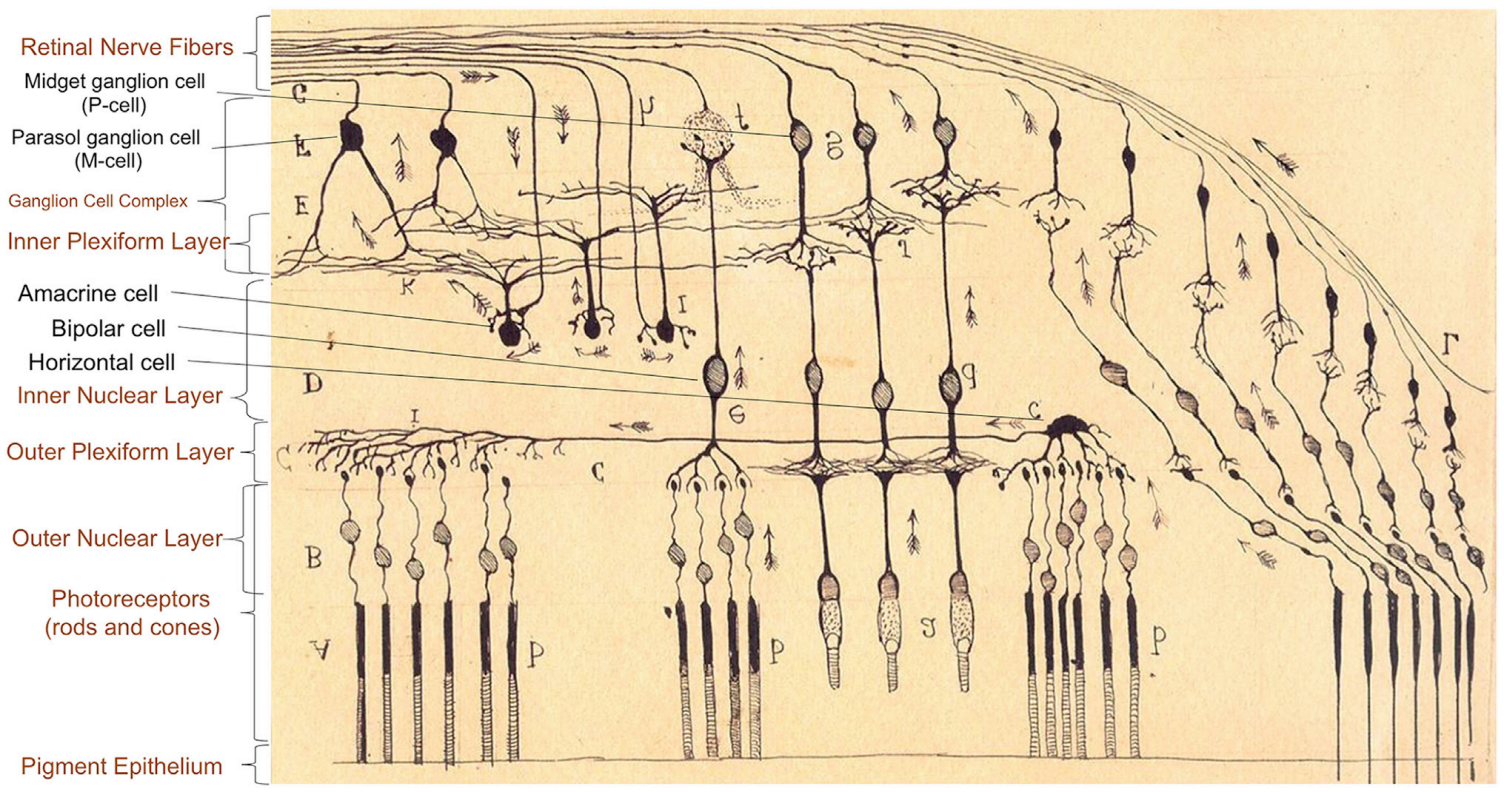
**Anatomy of retinal layers as described by Ramón y Cajal (9), Spanish neuroscientist pioneer in the investigation of the microscopy structure of the nervous system, including the retina, and Nobel Prize Awardee (1906)**. There are three layers of cells connected vertically (photoreceptors, bipolar cells, and ganglion cells) and horizontal interneurons modulating the signal conduction at two levels: horizontal cells in the conduction between photoreceptors (rods and cones) and bipolar cells in the outer plexiform layer, and amacrine cells between bipolar cells and ganglion cells in the inner plexiform layer. Large ganglion cells [retinal ganglion cells (RGC) projecting to the magnocellular pathway, M-cells] are specialized in motion detection and low spatial frequency achromatic contrast sensitivity. Smaller RGC with thinner axons (RGC projecting to the parvocellular pathway, P-cells) are concentrated in the central retina (macula), and they are responsible for visual acuity, color discrimination, and high spatial frequency chromatic/achromatic contrast sensitivity.

The retina derives embryologically from the neural tube and is part of the central nervous system. Because it is attached to the posterior surface of the ocular globe, the retina can be easily explored through the transparent media of the eye. The cellular architecture of the retina highly resembles the cerebral cortex with three layers of cells (instead of six) connected vertically by photoreceptors, bipolar cells, and ganglion cells, and horizontally by modulating interneurons. The interneurons group includes horizontal cells modulating the conduction between photoreceptors – rods and cones – and bipolar cells in the outer plexiform layer (OPL), and amacrine cells, modulating the conduction between bipolar cells and ganglion cells in the inner plexiform layer (IPL). Amacrine, bipolar, and horizontal cells are located in the inner nuclear layer (INL). The combination of the macular IPL, the macular ganglion cell layer (GCL), and the thin nerve fiber layer at the macula is referred to as ganglion cell complex (GCC) (Figure 1). Some OCT devices (e.g., Zeiss Cirrus^®^), however, do not include the retinal nerve fiber layer at the macula when assessing the GCC.

The visual information is highly processed and segregated in the retina and finally conducted using sub-populations of retinal ganglion cells (RGC), whose axons converge in the optic nerve. The classification of RGC sub-populations has evolved from the seminal morphological criteria of Ramón y Cajal (9) to more sophisticated criteria based on morphological, molecular, and genetic properties of the cells, particularly in murine models, with at least 25 RGC sub-populations identified so far (10). Based on their projections and functions, the classification of RGC can be simplified into four main sub-populations:

(a) Midget RGC (80%), projecting to the parvocellular layers of the lateral geniculate body (parvocellular pathway; P-cells). In this review, we refer to these RGC as P-cells, based on their anatomical projections in the lateral geniculate body.
(b) Parasol RGC (10%), projecting to the magnocellular layers of lateral geniculate body (magnocellular pathway; M-cells). In this review, we refer to these RGC as M-cells, based on their anatomical projections in the lateral geniculate body.
(c) Bistratified ganglion cells (10%), projecting to the koniocellular layers of lateral geniculate body (K pathway).
(d) Intrinsically photosensitive ganglion cells, projecting to suprachiasmatic nucleus.

M-cells, with wide retinal dendritic fields predominantly present in peripheral retinal regions, are specialized in motion detection and low spatial frequency achromatic contrast sensitivity. P-cells, with smaller dendritic fields and concentrated in the central retina (macula), are responsible for visual acuity, color discrimination, and high spatial frequency chromatic/achromatic contrast sensitivity (11). Axons from P-cells enter the temporal portion of the optic nerve in thinner nerve bundles, whereas axons from M-cells enter the inferior, superior, and nasal portions of the optic nerve in thicker bundles. Oligodendroglial cells myelinate the axons of M-cells and P-cells only after they exit the eye when crossing the lamina cribrosa (12).

### The Retina As a Biomarker in Neurodegenerative Disorders

Structural and functional changes of the retina are increasingly recognized as potential biomarkers for early diagnosis, prognosis, and progression of neurodegenerative disorders (13–15). The structure of the retina can be easily and non-invasively assessed with OCT, whereas retinal function can be evaluated with electrophysiological techniques, including visual evoked potentials (VEP) and electroretinography (ERG), and psychophysical methods, such as high and low contrast visual acuity, color, and movement perception. Pathological studies showed retinal damage in patients with Alzheimer disease (16), multiple sclerosis (17), idiopathic PD (18, 19), MSA (6), and mitochondrial neurodegenerative disorders (20).

In idiopathic PD and dementia with Lewy bodies (DLB), the most common synucleinopathies, visual symptoms are relatively frequent with ~80% of patients with PD reporting some visual-related problem such as double vision, difficulty to read despite adequate ocular refraction, gait freezing in narrow spaces, abnormal judgment of objects while walking, or visual hallucinations (21–24).

*In vivo* studies using OCT in non-human primate models of PD (MPTP-treated) (25) as well as in patients with PD have shown specific neurodegeneration of internal retinal layers. Initial reports showed atrophy of the peripapillary retinal nerve fiber layer (RNFL), especially in its temporal and temporal–inferior sectors (26, 27). Subsequent studies highlighted the presence of a specific profile of atrophy of inner retinal layers in the macula (28, 29). Recent publications have described flattening of the foveal pit affecting the foveal avascular zone of the macula (30, 31), a region with higher density of amacrine cells (32). Retinal damage in PD, as measured by OCT, has been associated with poorer visual function (33, 34), abnormal VEP and ERG (35), longer disease duration and higher symptomatic burden including dementia (36, 37), and visual hallucinations (38).

In line with OCT findings, histopathological evidence in animal models of PD showed a reduction in the number of dopaminergic cells (39–41). Postmortem studies of the retinas from patients with PD showed thinning of inner retinal layers, especially in the INLs, with intracellular and extracellular aggregates of αSyn in the GCL, IPL, and INL, not present in controls, suggestive of Lewy bodies (18). Another group showed αSyn deposition within retinal fibers penetrating the inner part of the retina, involving the RNFL, the GCL, and the IPL (19). This is in contrast to patients with MSA, in whom no retinal deposits of αSyn have been identified in spite of severely reduced density of RGC (6).

### Visual Abnormalities in MSA

In clinical practice, patients with MSA do not typically complain about specific visual problems, in contrast to the relatively frequent visual symptoms in patients with PD and DLB. When patients with MSA report eye-related symptoms, these are due to efferent (motor) visual system abnormalities, such as blepharospasm, blurry vision, or diplopia as a consequence of oculomotor abnormalities (e.g., excessive square jerks, mild vertical supranuclear gaze palsy, nystagmus, saccadic hypometria, impaired smooth pursuit, or visual oculocefalic reflex suppression) (42–46). The current consensus criteria on the diagnosis of MSA consider the presence of visual hallucinations not induced by drugs, a red flag against the diagnosis of MSA (47).

To have a statistical synthesis of the results of all retinal OCT studies in patients with MSA published to date, we performed a meta-analysis summarizing the differences reported in overall and sectorial RNFL thickness in MSA compared to healthy controls. Macular OCT measures were not included in the meta-analysis since acquisition protocols were highly variable across studies (Table 1). In contrast, acquisition and measurement protocols for RNFL thickness were consistent between the two most extensively used OCT apparatus (Heidelberg Spectralis^®^ and Zeiss Cirrus^®^) used in MSA studies. In fact, the agreement between those two specific devices to differentiate normal or abnormal RNFL thickness has been already demonstrated in ophthalmological diseases such as glaucoma (48).

**Table 1:** **Summary of retinal OCT studies in MSA**.

Reference	OCT device	Number of subjects	RNFL global (μm)	RNFL temporal (μm)	RNFL inferior (μm)	RNFL superior (μm)	RNFL nasal (μm)	TMT global (μm)	GCC global (μm)
Ahn et al. (50)	Spectralis^®^ (RNFL)/OPKO OTI^®^ (macula)	15 MSA	94.39 (13.92)	74.61 (12.71)	119.46 (21.44)*	114.75 (23.83)	68.79 (13.34)	268.78 (22.33)	–
		27 controls							
		23 MSA	102.15 (10.02)	79.32 (12.55)	132.26 (18.24)	124.58 (14.65)	72.43 (10.08)	273.74 (18.33)	–
		44 controls							

Mendoza-Santiesteban et al. (51)	Cirrus^®^	24 MSA	84.6 (5.0)*	59.7 (9.5)	108.1 (9.8)*	105.3 (11.6)	67.8 (5.9)	–	76.0 (6.3)*
		35 controls	89.8 (6.5)	62.0 (7.4)	117.9 (10.7)	109.4 (10.2)	70.7 (8.8)	–	80.6 (5.2)

Schneider et al. (56)	Cirrus^®^	12 MSA	–	–	–	–	–	267.5 (9.4)	66.7 (7.4)
		41 controls	–	–	–	–	–	277.5 (15.3)	72.4 (6.7)

Fischer et al. (54)	Spectralis^®^	12 MSA	93.18 (8.16)*	79.47 (16.17)	98.33 (14.81)	114.06 (16.1)	61.76 (13.46)*	228.82 (24.86)	–
		10 controls	97.20 (2.66)	75.30 (5.08)	123.7 (3.12)	117.8 (2.82)	71.10 (1.52)	232.30 (10.24)	–

Albrecht et al. (52)	Spectralis^®^	19 MSA	93.79 (1.92)	72.37 (3.45)	118.4 (4.16)	115.9 (3.7)	68.18 (2.23)	308.2 (4.13)	96.08 (1.95)
		35 controls	99.13 (1.59)	73.89 (2.02)	126.7 (3.06)	121.6 (2.78)	71.99 (2.34)	317.6 (2.69)	98.7 (1.60)

Pula et al. (55)	Spectralis^®^	5 MSA	100 (11)	–	–	–	–	3 mm/6 mm	–
								314 (12)*/285 (15)	
		27 controls	98 (9)	–	–	–	–	339 (17)/295 (17)	–

Fischer et al. (53)	Spectralis^®^	10 MSA	91.30 (1.45)*	83.40 (3.25)	114.15 (3.43)	111.05 (3.04)	61.70 (1.63)	234.20 (5.14)	–
		10 controls	97.2 (1.45)	75.30 (3.25)	123.7 (3.43)	117.8 (3.04)	71.10 (1.63)	232.30 (5.14)	–

*p < 0.05 MSA versus controls.

*OCT, optical coherence tomography; MSA, multiple system atrophy; RNFL, peripapillary retinal nerve fiber layer thickness; TMT, total macular thickness; GCC, ganglion cell complex (ganglion cell layer + inner plexiform layer) thickness; global, average of all measurements; temporal, inferior, superior and nasal, sectors of RNFL*.

## Methods

The meta-analysis was prepared according to the preferred reporting items for systematic reviews and meta-analyses guidelines (49). Articles on OCT and MSA were identified by searches of PubMed through January 1, 2017. We included only articles in English. The following search terms were used: “multiple system atrophy,” “MSA,” “Shy-Drager,” “striatonigral degeneration,” “olivopontocerebellar,” “autonomic failure,” “optical coherence tomography,” “OCT,” and “retina.” We also reviewed the reference lists of the retrieved articles. We did not include unpublished data or data from abstracts.

For the meta-analysis, articles were evaluated independently by two reviewers (Iñigo Gabilondo and Jose-Alberto Palma) who extracted the following data from each study: first author, year of publication, OCT device type, study participants (MSA and controls), and OCT results (mean and standard deviation) on the thickness (in µm) of the following retinal areas: average RNFL, temporal RNFL, inferior RNFL, superior RNFL, and nasal RNFL. Case reports were excluded.

### Statistical Analysis

We used combined mean difference as a common measure of association between MSA and retinal thickness. The pooled difference in the thickness of specific retinal areas between MSA and controls and 95% confidence intervals were obtained by using a random-effects model. We used a random-effects model rather than a fixed-effects model because of the high likelihood of heterogeneity between study variance. The heterogeneity of effect size estimates across studies was described with the *I*^2^ index (with values of 25, 50, and 75% considered low, moderate, and high, respectively). Sensitivity analysis was performed using the leave-one-out approach. Analyses were performed with Review Manager 5.3 (Cochrane Collaboration, Nordic Cochrane Center, Denmark). *p* < 0.05 was considered as statistically significant, indicating significant thinning in patients with MSA versus controls.

## Results

### Meta-Analysis Results

**Figure 2:**
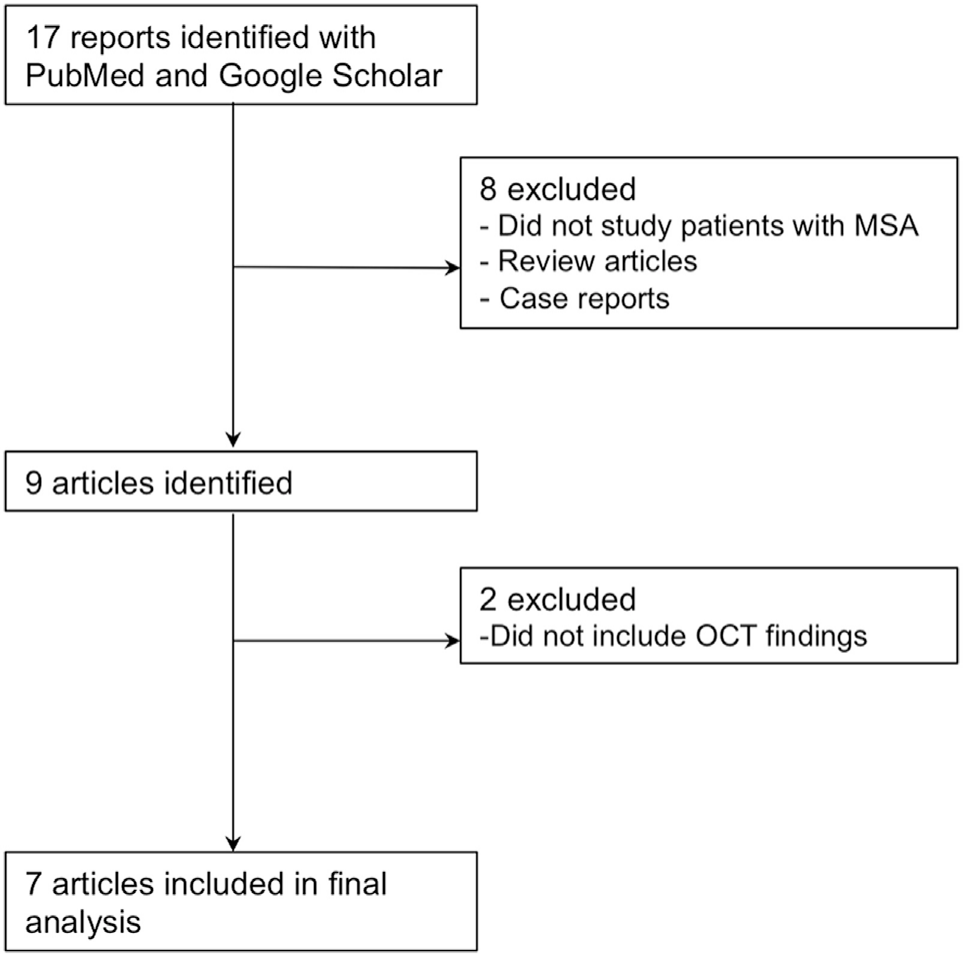
**Flowchart of study selection for the meta-analysis**.

As shown in Figure 2, the primary search strategy yielded 15 articles of which 7 met the inclusion criteria. Table 1 shows the characteristics of the seven identified studies.

#### Retinal Nerve Fiber Layer in MSA

**Figure 3:**
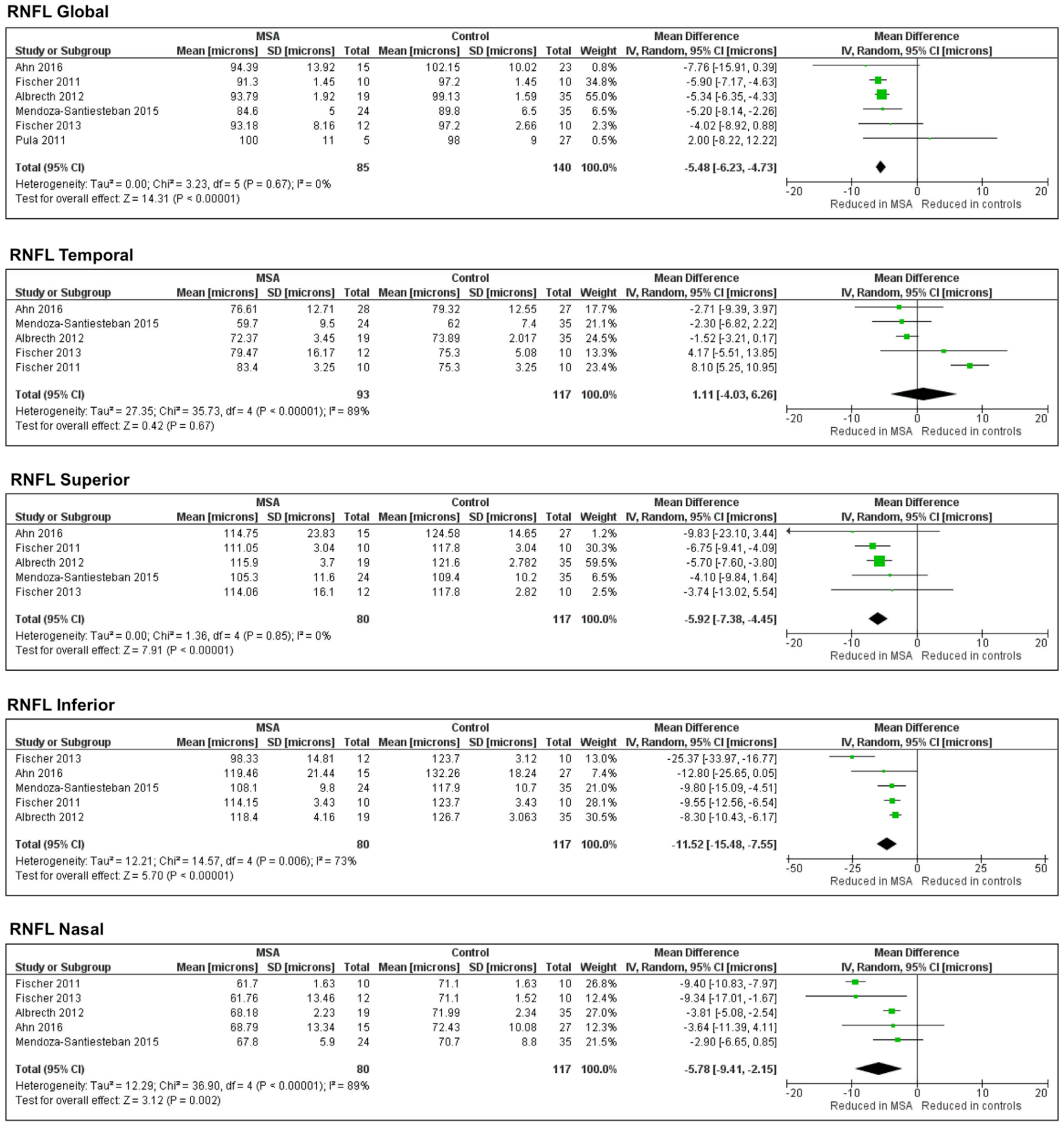
**Forest plots showing the pooled difference in the average thickness of the retinal nerve fiber layer in global and specific quadrants of multiple system atrophy (MSA) versus controls**.

The pooled difference in the average RNFL thickness between controls and MSA was −5.48 μm (95% CI, −6.23 to −4.73; *p* < 0.0001), indicating a significant thinning in controls versus MSA. The pooled results showed significant thinning in MSA versus controls in all the specific RNFL quadrants, except in the temporal RNFL quadrant, where the thickness between MSA and controls was not different [pooled difference between controls and MSA was 1.11 µm (95% CI, −4.03 to 6.26; *p* = 0.67)] (Figure 3).

#### Sensitivity Analysis and Publication Bias

Of the seven studies included in this meta-analysis, two (50, 51) used Cirrus^®^. The remaining five (52–56) used Spectralis^®^. To clarify the potential effect on the pooled results caused by the different devices, we conducted a sensitivity analysis to explore potential sources of heterogeneity. After excluding the two studies that used Cirrus^®^, all the results remained unchanged. Because of the small number of study retrieved, no publication bias analysis was performed.

### Literature Review Results

#### Electrophysiology in MSA

The initial evidence of the dysfunction of visual pathways in MSA was obtained with visual electrophysiology studies (57, 58). These found significant inter-eye difference in contrast sensitivity and latency delay in PD, which were not present in patients with MSA. One of these studies specifically evaluated retinal integrity in six patients with MSA, 12 patients with PD, and 33 healthy controls using flash and pattern ERG (in addition to VEP and psychophysical contrast thresholds, contrast discriminations and reaction times) (59). This study disclosed ERG abnormalities in patients with MSA, although much less severe than in patients with PD. Another study including six patients with MSA and 12 with PD showed that the chromatic pattern-reversal ERG is spared in MSA, in contrast to PD (60).

#### OCT in MSA

The first OCT study in patients with MSA (53) included 10 patients with MSA and 10 age-matched controls. The average peripapillary RNFL (RNFL) (in circular B-scans centered in optic disk) and the total retinal thickness (in two linear B-scans at the foveola) were studied. The investigators found that the global RNFL thickness was significantly reduced in MSA patients compared to controls, particularly in the nasal quadrant. No differences in total retinal thickness were found.

Another study published shortly after (55) included five patients with MSA-C and 27 healthy controls. The study measured average RNFL thicknesses and global and sectorial total macular thickness, finding that global macular thickness was significantly reduced in MSA-C versus controls only in the 3-mm (but not in the 6-mm) diameter ring as well as in the temporal sector of the 6-mm macular ring. Unfortunately, no information on specific sectors of the RNFL was included in this study.

In 2012, Albrecht and colleagues (52) performed OCT studies in 19 patients with MSA, 40 patients with PD, 10 with corticobasal degeneration, 15 with progressive supranuclear palsy (PSP), and 35 controls. The results showed significant atrophy of the peripheral macula in patients with MSA compared to controls, but no differences in total or central macular or RNFL thickness. This study evaluated for the first time differences in thickness of deeper retinal layers of the macula using a semi-automatically segmented single B-scan situated in the middle of the fovea. These included the GCL and IPL complex (GCC + IPL, also known as the GCC), the INL, the OPL and the outer nuclear layer (ONL). No differences between MSA and controls were found in any of these layers.

Another study in 2013 evaluated 12 patients with MSA and 10 age-matched healthy controls (54). The study did not find differences in foveal thickness or global RNFL. However, the nasal RNFL was significantly thinner in MSA. In addition, the authors found no association between any OCT measurements, and visual field abnormalities, disease severity as measured by the United Multiple System Atrophy Rating Scale (UMSARS), and disease duration. It is unclear if, in this study, the authors included data of patients from their 2011 study (53).

In 2014, Schneider and colleagues (56) evaluated retinal damage in the macula and its layers using OCT in MSA (*n* = 12), PSP (*n* = 16) and PD (*n* = 65) patients, and 41 controls. They found no differences in the RNFL, GCC, and the INL in patients with MSA versus controls. Interestingly, they did find a significant thickening in the ONL and OPL of MSA versus controls, whereas in patients with PSP the ONL was thinner and the OPL was ticker than controls. The authors suggested that the ONL/OPL ratio could be useful to distinguish MSA from PSP, with high sensitivity (88%) and specificity (91%). This same study found no association between retinal thickness and neurological disability scores in MSA.

In 2015, we (51) published a cross-sectional study including 24 MSA, 20 PD patients, and 35 healthy controls. We found no differences in best-corrected high-contrast visual acuity and color vision between MSA and controls. OCT showed thinner RNFL (average and inferior quadrant) and thinner GCC in the macular cube in MSA patients. We also observed a tendency toward thinner GCC globally and thinner RNFL for all quadrants, especially for temporal RNFL in patients with PD compared to MSA. This preferential atrophy of the temporal RNFL quadrant in PD was also found when compared to controls.

Ours (51) was the first study to document progressive longitudinal changes in the retina in patients with MSA, as 13 of the initial 24 MSA patients were also followed up overtime with two to seven visits for a mean of 12 months (maximum follow-up of 26 months). We observed a rate of thinning in the RNFL of −3.72 μm (−4.32%) per year. This RNFL reduction rate was higher than the one of healthy subjects (−0.33 μm/year) and also notably higher than the one reported in multiple sclerosis (−2.0 μm/year). The macular GCC also thinned, although to a much lesser rate of −1.8 μm (−2.52%) per year. Longer follow-up periods were associated with more intense thinning of the RNFL and GCC. In an attempt to open the door to using OCT in clinical trials of MSA, we also estimated the required number of patients for clinical trials in order to use the RNFL thickness as an objective outcome measure.

The most recent study using OCT (50) analyzed the RNFL in 15 patients with MSA and 27 controls, and total macular thickness in 23 MSA and 44 controls. This study showed a significant thinning of the RNFL and total retinal thickness in outer superior macular sectors in patients with MSA. Total macular thickness in patients with MSA was associated with their UMSARS and Global Disability Score. For unclear reasons, the authors used different OCT devices for the acquisition of the RNFL thickness (Spectralis^®^) and the macular thickness (OPKO OTI^®^).

The reviewed studies of OCT in MSA have some common limitations:

(a) The highest sample size so far has been 24 MSA patients (51), which is relatively low, although MSA is a rare disease.
(b) Only one study (51) measured longitudinal changes over time; all other studies were cross-sectional with no follow-up.
(c) The use of different OCT devices (Cirrus^®^, Spectralis^®^, OPKO OTI^®^) and algorithms may lead to heterogeneous results. In this regard, the acquisition protocol of macular measurements (e.g., image resolution, number and dimension of slices, analyzed areas from those slices, layers analyzed, and segmentation methods) varied considerably among studies, and in some studies certain macular measurements were not reported.
(d) The statistical analysis of the OCT results was markedly different: most studies averaged the results of both eyes (51, 54–56); two considered the results of each eye as independent values (50, 52), whereas one study used only the results of the right eye (53).

## Discussion

Although afferent visual symptoms are uncommon in patients with MSA, OCT and electrophysiological studies support the presence of retinal abnormalities in these patients.

The retinal damage in patients with MSA appears to follow a different pattern to that observed in those with PD. While in PD patients the atrophy of temporal RNFL sectors and internal retinal layers (i.e., GCC) at the parafoveal region are prominent, in patients with MSA the inferior, superior and nasal RNFL sectors are more affected than in PD.

**Figure 4:**
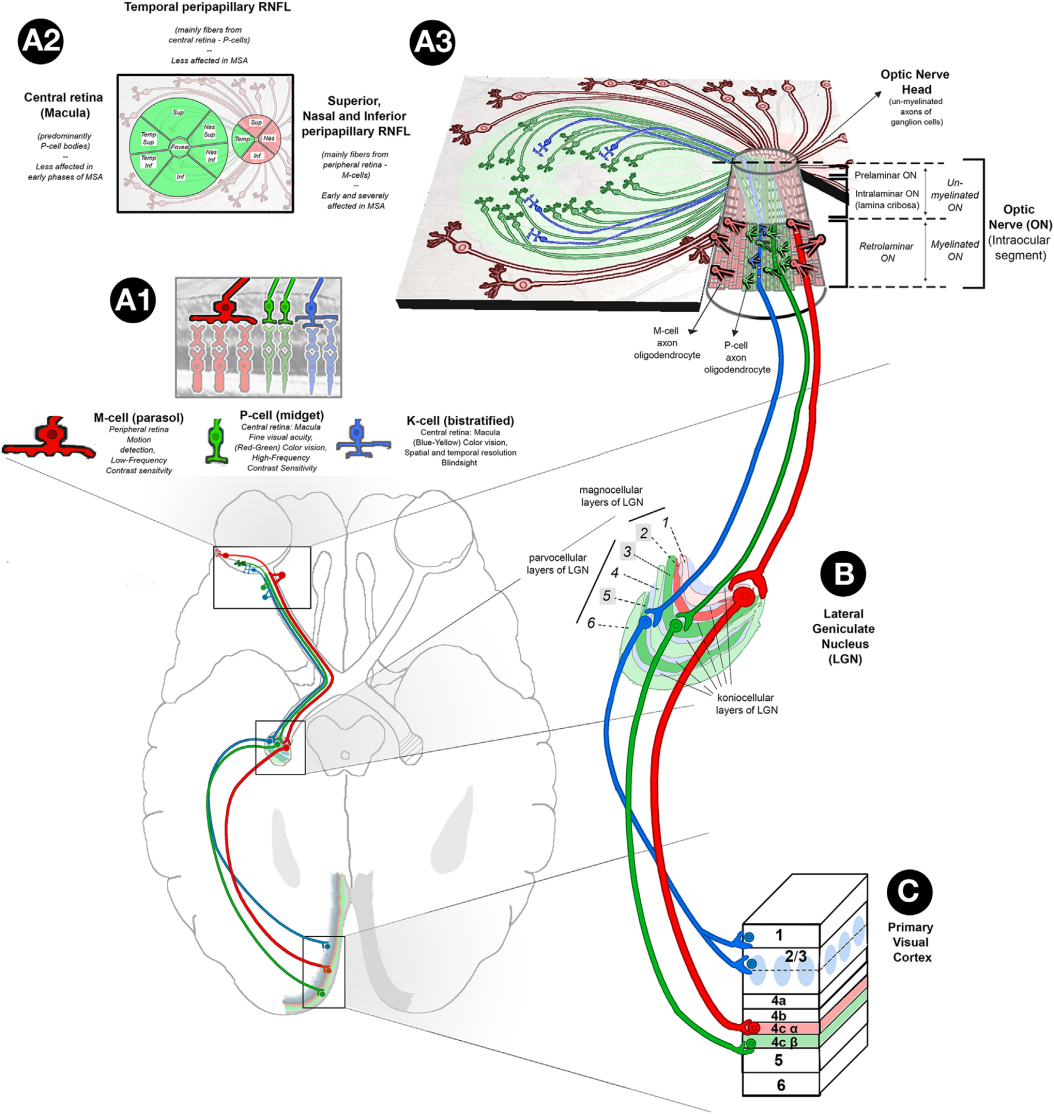
**Retinal abnormalities in MSA**. **(A1)** Based on their morphology, functions, and projections to specific layers of LGN, there are three main types of ganglion cells in the retina: (1) parasol cells or M-cells (represented in red) (10%): their cell bodies are predominantly located in the peripheral retina, and their axons project through RNFL to sup, nas, and info sectors of the ON head, ultimately reaching the magnocellular layers of the LGN; M-cells are responsible for movement discrimination and low-frequency contrast sensitivity; (2) midget ganglion cells, or P-cells (represented in green) (80%): their cell bodies are predominantly located in the central retina (macula), and their axons project through the papillomacular bundle of the RNFL to the temp sector of the ON head, reaching parvcellular layers of the LGN; their function has been related to fine visual acuity (red–green) color vision and high-frequency contrast sensitivity; and (3) bistratified cells, or K-cells (represented in blue) (10%): their distribution in the retina is similar to P-cells, and their axons synapse with koniocellular layers of the LGN; their function is related to blue–yellow color vision, different aspects of spatial and temp resolution and blind sight. **(A2)** Retinal map of the distribution of P-cell (green color) and M-cell (red color) bodies and axons. **(A3)** Tridimensional representation of retina P-, M-, and K-cell fibers and their organization in the intraocular ON (info center): the intraocular ON is the first segment of the ON after ON head, in which RNFL axons penetrate the neural retina, choroid, and sclera to form the extraocular ON. The intraocular ON is divided from proximal to distal in preliminar, intralaminar, and a retrolaminar portions. The intralaminar portion contains the lamina cribrosa, a multilayered network of collagen fibers that insert into the scleral canal wall. When un-myelinated axons of P, K, and M-ganglion cells reach the lamina cribrosa, they become myelinated by the myelin sheath of ON oligodendrocytes, each of them covering several ganglion cell axons. According to current evidences on optical coherence tomography, in MSA, sup, nas, and info sectors of peripapillary RNFL are affected early and severely while temp sectors of peripapillary RNFL and central macular ganglion cell layer are relatively spared. This finding suggests a specific pattern of retina damage in MSA in which M-cells are specifically affected. This hypothesis is physiopathologically plausible, since MSA is a primary oligodendropathy and M-cells with their bigger axons may require higher myelination support from oligodendrocytes. **(B)** The LGN has layers of magnocellular cells and parvocellular cells that are interleaved with layers of koniocellular cells. In humans, the LGN is normally described as having six distinctive layers. The inner two layers (1 and 2) are magnocellular layers, while the outer four layers (3, 4, 5, and 6) are parvocellular layers. Koniocellular cells are located in additional set of layers found ventral to each of magnocellular and parvocellular layers. Layers 2, 3, and 5 receive inputs from ganglion cells of ipsilateral retina (highlighted in brighter colors), and layers 1, 4, and 6 receive ganglion cell axons from contralateral retina that crossed the chiasm. **(C)** Primary Visual Cortex (V1). P-cells project to parvocellular layers of the LGN and on to layer 4Cβ of V1. M-cells project to magnocellular layers of the LGN and on to layer 4Cα of V1. K-cells project to koniocellular layers of the LGN and on to the cytochrome oxidase-expressing patches (or blobs) of layer 2/3 and to layer 1. Abbreviations: sup, superior; nas, nasal; temp, temporal; info, inferior; MSA, multiple system atrophy; LGN, lateral geniculate nucleus; RNFL, retinal nerve fiber layer; ON, optic nerve.

**Figure 5:**
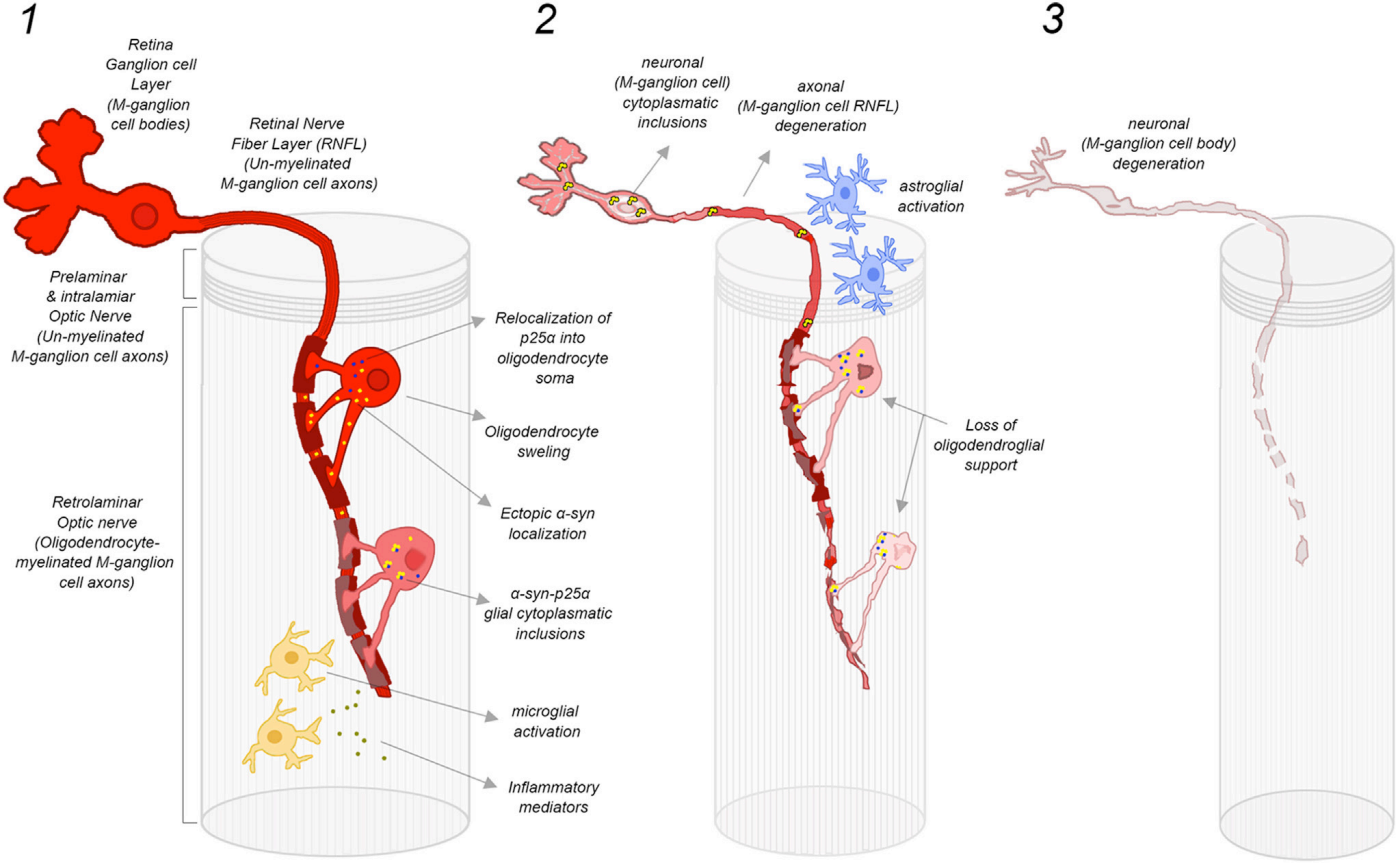
**Damage mechanisms and progression of retinal M-ganglion cell degeneration in MSA**. (1) Early stage of MSA: oligodendrocytes located in the preliminary ON provide myelin sheath to several P-cell and M-cell axons. In the case of M-cells, the level of myelination support is particularly prominent since their axons are larger. ON oligodendrocytes in MSA are potentially susceptible to follow the same cellular pathological cascade that has been proposed for brain oligodendrocytes, with an initial relocalization of p25α into oligodendrocyte soma and ectopic localization of αSyn, leading to a progressive oligodendrocyte swelling. The formation of α-syn-p25α glial inclusions may induce the activation of microglia and the release of inflammatory factors, which further contributes to oligodendrocyte and myelin degeneration. This early oligodendrocyte damage may induce also early injury of M-cell axons favoring the early atrophy of superior, nasal, and inferior sectors of ON head RNFL; (2) Advanced stage of MSA: the severe degeneration of oligodendroglia leads to a loss of oligodendroglial support, which promotes the degeneration of axons of M-ganglion cells. In addition, there is a liberation of misfolded αSyn by oligodendrocytes to extracellular space, which may be taken by adjacent neurons to form misfolded αSyn inclusions within axons and cell bodies of M-cells. Misfolded αSyn inclusions within M-cells further promote neuronal dysfunction and neurodegeneration, with a reactive activation of local ON astroglia. In this phase, there is a severe damage of M-cell axons in the retina (superior, nasal, and inferior sectors of ON) that may also involve to a lesser extent P-cell axons (atrophy of temporal sector of ON RNFL) and their cell bodies (macular GCL atrophy); and (3) End-stage MSA: there is a severe degeneration of M-cell axons and cell bodies that extend also to P-cell axons and bodies, reflected in the retina as a widespread atrophy of peripapillary RNFL (still more prominent in superior, nasal, and inferior sectors) and macular GCL. Abbreviations: MSA, multiple system atrophy; ON, optic nerve; αSyn, α-synuclein; RNFL, retinal nerve fiber layer; GCL, ganglion cell layer. Figure inspired by Ref. (1).

The dissimilarities between PD and MSA patients at the clinical and retinal level could be explained by differences in the preferential damage of P-cells versus M-cells (51). P-cells predominate in the central macular region (where the macular GCC is measured), their axons project to the temporal portion of the retinal nerve fiber layer and they are highly related to color discrimination, visual acuity, central visual field sensitivity, and contrast sensitivity for high spatial frequencies. On the other side, axons from M-cells (with cell bodies situated in peripheral macula and retina) are located in the superior, nasal, and inferior regions around the optic nerve (where the RNFL is measured). These M-cells relay information about achromatic vision, peripheral visual field sensitivity, motion detection, and contrast sensitivity for low spatial frequencies. The fact that MSA patients typically have normal visual acuity and color vision in combination with inferior RNFL atrophy may indicate that, in MSA, M-cells are more affected than P-cells. This is in contrast to PD patients, who have preferential atrophy of temporal RNFL sectors and higher central macular GCC atrophy (compared to MSA), and is in keeping with their relatively frequent complains of visual problems, such as decreased visual acuity, impaired color discrimination, defective motion perception, and visual hallucinations (Figure 4). The hypothesis of a preferential injury of P-cells in PD and M-cells in MSA is still unproven, but it may be related to a predominant damage of optic nerve axons in MSA that being especially coarse and myelinated (like those from M-cells) require high support from oligodendrocytes (Figure 5).

## Conclusion

**Figure 6:**
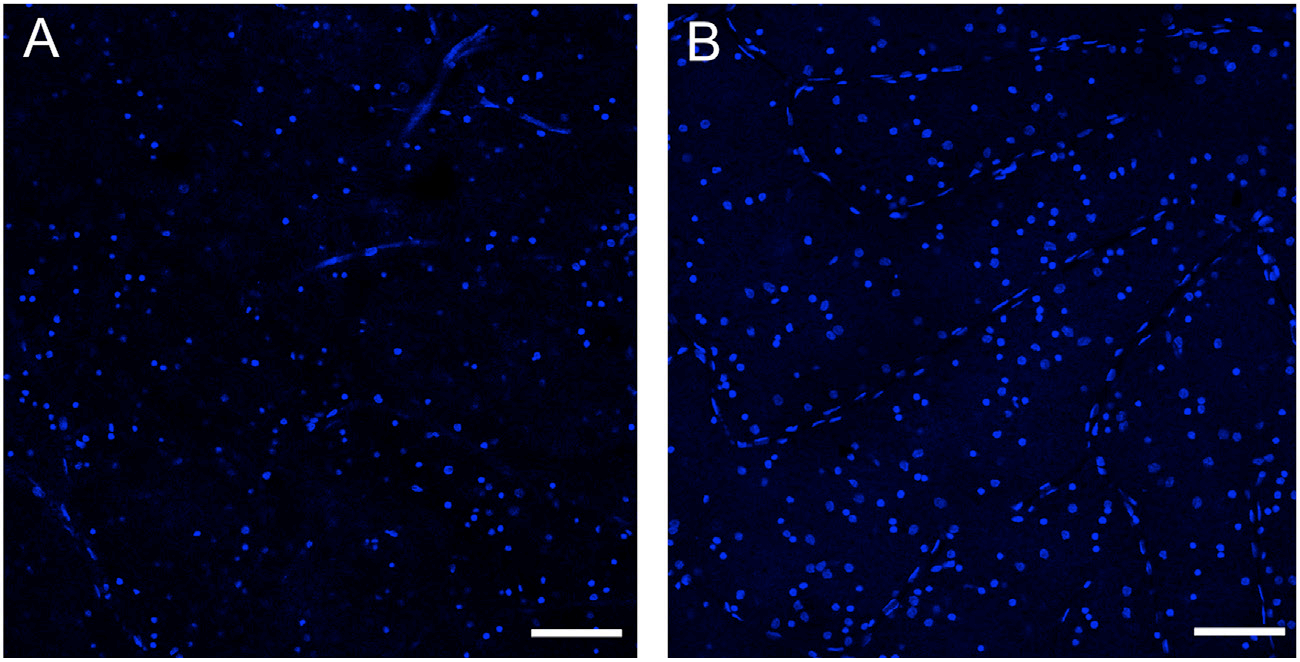
**Loss of retinal ganglion cells in the peripheral retina of a patient with multiple system atrophy (MSA)**. Confocal images of representative areas of whole-mounted retinae (superior–temporal area of the far peripheral retina; distance from the ora serrata: 1–5 mm) labeled with the blue fluorescent Hoechst marker of a patient with MSA **(A)** and an age-matched normal subject **(B)**. Scale bar is 100 µm. The number of ganglion cells is markedly reduced in MSA compared to the control. See Ref. (6) for additional information.

Multiple system atrophy is a rare, adult-onset fatal synucleinopathy driven by a primary dysfunction of CNS oligodendrocytes. While efferent visual or oculomotor symptoms are relatively common in MSA patients, most MSA patients rarely report afferent visual symptoms. Despite the paucity of symptoms, our meta-analysis shows that patients with MSA have significantly decreased RNFL in all, except in the temporal quadrant. Two publications also showed that these retinal thinning worsens with disease progression and severity (50, 51). Pathological confirmation of reduced peripheral RGC in patients with MSA has been recently reported (6) (Figure 6).

The retinal damage in MSA patients follows a different pattern to that observed in those with PD. Patients with MSA have more prominent atrophy of inferior sectors of the RNFL and more preserved central macular GCC. Patients with PD have more atrophy in the temporal RNFL and the GCC layers of the central–parafoveal macula. We hypothesize that in patients with MSA there is predominant damage of large myelinated optic nerve axons like those from the M-cells. These large axons may require higher support from oligodendrocytes. In contrast, in patients with PD, P-cells appear to be more affected. Further studies with pathological confirmation are required to validate these findings.

## Author Contributions

CM-S, IG, and J-AP: Study concept and design, acquisition of data, analysis and interpretation, critical revision of the manuscript for important intellectual content, study supervision. LN-K and HK: acquisition of data, analysis and interpretation, critical revision of the manuscript for important intellectual content.

## Conflict of Interest Statement

The authors declare that the research was conducted in the absence of any commercial or financial relationships that could be construed as a potential conflict of interest.

## Abbreviations

αSyn, α-synuclein; DLB, dementia with Lewy bodies; ERG, electroretinography; GCC, ganglion cell complex; GCL, ganglion cell layer; INL, inner nuclear layer; IPL, inner plexiform layer; MSA, multiple system atrophy; MSA-C, multiple system atrophy-cerebellar; MSA-P, multiple system atrophy-parkinsonian; OCT, optical coherence tomography; ONL, outer nuclear layer; OPL, outer plexiform layer; PD, Parkinson disease; PSP, progressive supranuclear palsy; RGC, retinal ganglion cells; RNFL, peripapillary retinal nerve fiber layer; UMSARS, united multiple system atrophy rating scale; VEP, visual evoked potentials abnormalities.
